# The Regulatory Role of MeAIB in Protein Metabolism and the mTOR Signaling Pathway in Porcine Enterocytes

**DOI:** 10.3390/ijms19030714

**Published:** 2018-03-02

**Authors:** Yulong Tang, Bie Tan, Guangran Li, Jianjun Li, Peng Ji, Yulong Yin

**Affiliations:** 1 Laboratory of Animal Nutritional Physiology and Metabolic Process, Key Laboratory of Agro-ecological Processes in Subtropical Region, National Engineering Laboratory for Pollution Control and Waste Utilization in Livestock and Poultry Production, Institute of Subtropical Agriculture, Chinese Academy of Sciences, Changsha 410125, China; tangyulong@isa.ac.cn (Y.T.); liguangran@anschina.cn (G.L.); jianjunli@isa.ac.cn (J.L.); 2 Hunan Co-Innovation Center of Animal Production Safety (CICAPS), Changsha 410128, China; 3 Department of Nutrition, University of California, Davis, CA 95616, USA; penji@ucdavis.edu

**Keywords:** sodium-coupled neutral amino acid transporter 2, mTOR, protein turnover, porcine enterocytes

## Abstract

Amino acid transporters play an important role in cell growth and metabolism. MeAIB, a transporter-selective substrate, often represses the adaptive regulation of sodium-coupled neutral amino acid transporter 2 (SNAT2), which may act as a receptor and regulate cellular amino acid contents, therefore modulating cellular downstream signaling. The aim of this study was to investigate the effects of MeAIB to SNAT2 on cell proliferation, protein turnover, and the mammalian target of rapamycin (mTOR) signaling pathway in porcine enterocytes. Intestinal porcine epithelial cells (IPEC)-J2 cells were cultured in a high-glucose Dulbecco’s modified Eagle’s (DMEM-H) medium with 0 or 5 mmoL/L System A amino acid analogue (MeAIB) for 48 h. Cells were collected for analysis of proliferation, cell cycle, protein synthesis and degradation, intracellular free amino acids, and the expression of key genes involved in the mTOR signaling pathway. The results showed that SNAT2 inhibition by MeAIB depleted intracellular concentrations of not only SNAT2 amino acid substrates but also of indispensable amino acids (methionine and leucine), and suppressed cell proliferation and impaired protein synthesis. MeAIB inhibited mTOR phosphorylation, which might be involved in three translation regulators, *EIF4EBP1*, *IGFBP3*, and *DDIT4* from PCR array analysis of the 84 genes related to the mTOR signaling pathway. These results suggest that SNAT2 inhibition treated with MeAIB plays an important role in regulating protein synthesis and mTOR signaling, and provide some information to further clarify its roles in the absorption of amino acids and signal transduction in the porcine small intestine.

## 1. Introduction

Amino acid transporters are membrane transport proteins; their major role is transporting amino acids and modulating gene expression and the signal transduction pathway by sensing amino acid levels [1]. The SLC38 family of transporters represents a main branch of solute carrier families in mammals, and the transporters can be subdivided into two groups, namely system A and system N [2,3]. Sodium-coupled neutral amino acid transporter 2 (SNAT2) has a very broad tissue distribution profile and is characterized as a system A transporter, which plays various roles in different tissues and has dual transport/receptor functions [4,5]. α-Methylaminoisobutyric acid (MeAIB), a system A substrate, suppresses the expression of SNAT2 in cells and has been used as a means of inhibiting the uptake of natural system A substrates [2].

SNAT2 is well documented in human cancer cells and skeletal muscle myoblast cells. Currently it has been demonstrated to sense intracellular anabolic amino acid levels and then regulate the amino acid signaling pathways influencing protein turnover and cell growth [4,5,6,7,8]. Several studies showed that SNAT2 has a low level of expression in complete media, but its activity could be enhanced by withdrawing amino acids, which provide evidence of SNAT2’s ability to regulate amino acid homeostasis as a transporter [9,10]. Potential regulatory mechanisms of SNAT2 in amino acid nutrition has demonstrated it is involved in eukaryotic initiation factor 2 phosphorylation, increased gene transcription, and internal ribosome entry site-mediated translation [9,10]. The intestinal porcine epithelium comprises a large surface area lined by a single layer of columnar intestinal epithelial cells with the expression of a variety of transporters in apical and basolateral membranes; these transporter proteins play complex and interactive roles in controlling the absorption and metabolism of amino acids [11,12]. We have previously cloned and described the SNAT2 gene in the small intestine of piglets, which is highly evolutionarily conserved in humans [4]. This study was conducted to investigate the role of the SNAT2 transporter after the addition of MeAIB in cell growth, protein turnover, and its related mTOR signaling in the intestinal porcine epithelial IPEC-J2 cells.

## 2. Results

### 2.1. SNAT2 Inhibition Decreased Cell Growth and Intracellular cAMP Concentration in IPEC-J2 Cells 

**Figure 1:**
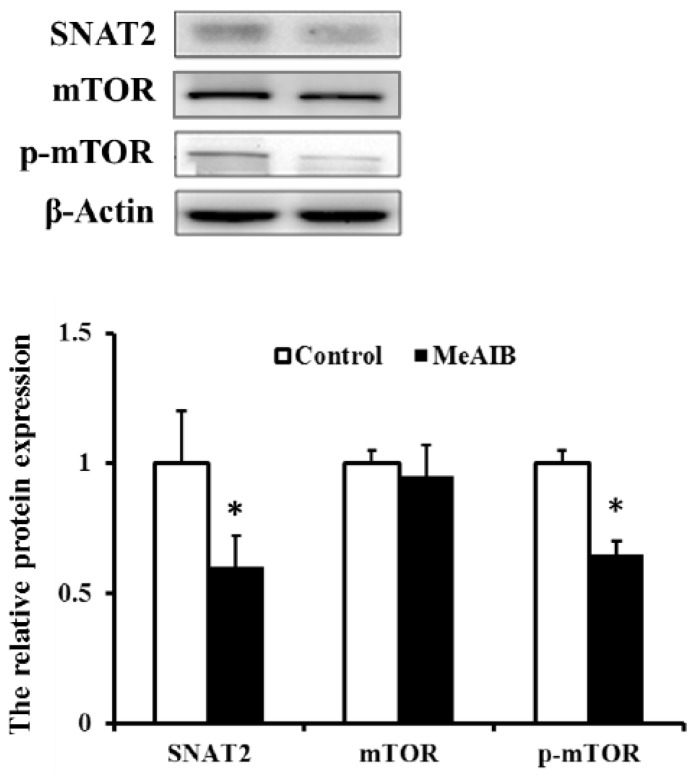
MeAIB induces the inhibition of SNAT2 and mTOR phosphorylation in IPEC-J2 cells. Data are expressed as mean ± Standard Deviation (SD), *n* = 4 independent experiments. * *p* < 0.05 versus control treatment.

Immunoblotting of IPEC-J2 treated with MeAIB caused a significant decrease in SNAT2 expression (*p* < 0.05). mTOR phosphorylation was more strongly inhibited compared with the control group (*p* < 0.05) (Figure 1). There was no change in total mTOR abundance.

**Figure 2:**
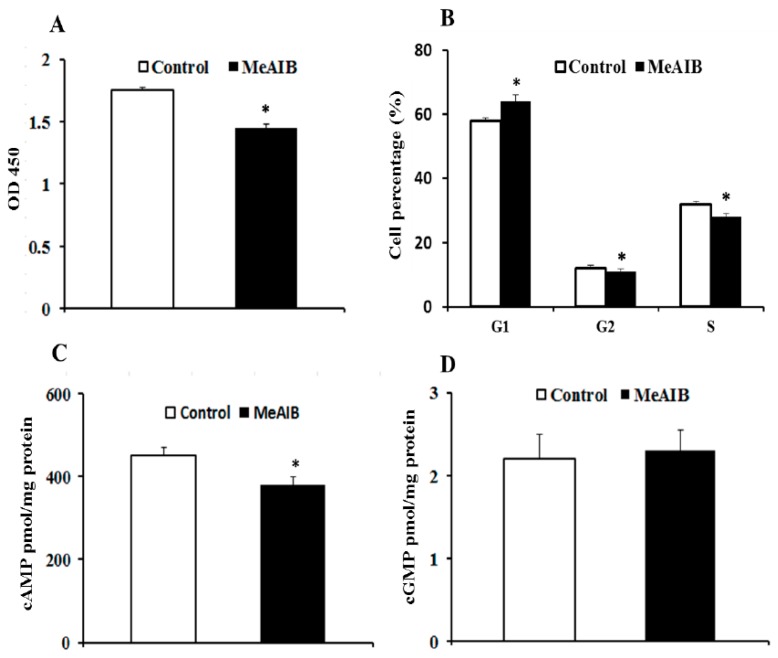
Effects of MeAIB on cell proliferation (**A**), cell cycle (**B**), intracellular cAMP (**C**), and cGMP (**D**) concentrations in IPEC-J2 cells. Cell proliferation was determined using the Cell Counting Kit-8 (CCK-8, Dojindo Molecular Technologies, Inc., Rockville, MD, USA) at 450 nm. Cell cycles were analyzed using propidium iodide DNA staining and Flow Cytometry. Intracellular cAMP and cGMP were determined by immunoassay. Data are expressed as mean ± SD, *n* = 4 independent experiments. * *p* < 0.05 versus control treatment.

MeAIB significantly reduced IPEC-J2 cell growth, reaching statistical significance at two days post-MeAIB addition (*p* < 0.05) (Figure 2A). IPEC-J2 cells were subjected to cell cycle analysis using flow cytometry after treating with MeAIB for 48 h, and Figure 2B showed that the number of cells in the G1 phases increased and the proportion of cells in the G2 and S phases substantially decreased (*p* < 0.05).

The intracellular cGMP level of IPEC-J2 treated with MeAIB did not show a significant effect (*p* > 0.05); by contrast, the cells displayed a significantly lower concentration of cAMP when cultured in the presence of MeAIB, compared with the control group (Figure 2C,D).

### 2.2. MeAIB Affects Protein Synthesis, but Not Degradation in IPEC-J2 Cells

**Figure 3:**
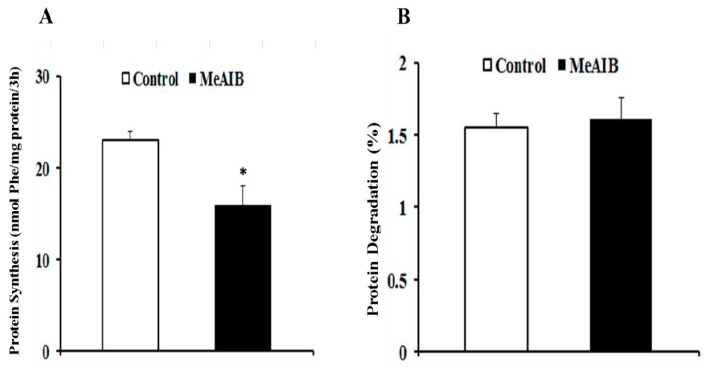
Effect of IPEC-J2 cells’ incubation with MeAIB on protein synthesis (**A**) and degradation (**B**). Protein synthesis (nmol Phe/mg) and protein degradation (%) measured using (3H) labeled phenylalanine. Data are expressed as mean ± S, *n* = 4 independent experiments. * *p* < 0.05 versus control treatment.

Addition of MeAIB to the culture medium resulted in a decrease of (^3^H)-phenylalanine incorporation in proteins, which reduced the isotope uptake by approximately 30% (Figure 3A) (*p* < 0.05) compared with the control group, while there was no significant effect on protein degradation (*p* > 0.05) (Figure 3B).

### 2.3. Involvement of MeAIB in Decreasing Amino Acid Transport

The inhibition of total expression of the SNAT2 protein (approximately 50%) by MeAIB led to a significant reduction of some intracellular amino acids, including l-Glu 5.7-fold, l-Pro 2.67-Fold, l-Met 2.65-fold, l-Ser 2.31-fold, l-His 2.45-fold, l-Gly 2.30-fold, l-Asp 1.88-fold and l-Leu 1.22-fold *(p* < 0.05). There were no effects on other amino acids (Table 1).

### 2.4. Evaluation of Transcriptional Expression of Genes Related to mTOR Signaling Pathway in IPEC-J2 Cells

Among the 84 simultaneously detected genes related to the mTOR signaling pathway, 21 exhibited significant changes in expression (Table 2). The level of expression of 11 genes, including *AKT1*, *RPS6KA4*, *DDIT4*, *EIF4EBP1*, *IGFBP3*, *INS*, *PIK3CG*, *PRKCB*, *RPS6KA1*, *RPTOR*, and *ULK1*, was upregulated, with most of them belonging to the mTOR upstream regulator and having the role of both positive and negative regulation. In contrast, 10 genes, *CAB39*, *DDIT4L*, *IRS1*, *KRAS*, *PIK3R2*, *RPS6KA5*, *VEGFB*, *RRAGA*, *VEGFC*, and *YWHAQ*, were significantly downregulated in IPEC-J2. Four of them are not translation regulators for mTOR, and for the other six regulated genes (*CAB39*, *IRS1*, *VEGFB*, *RRAGA*, *VEGFC*, and *YWHAQ*), three of them (*IRS1*, *RRAGA*, and *YWHAQ*) existed mTOR upstream and three were located on the mTOR downstream.

## 3. Discussion

Amino acids not only serve as precursors for the synthesis of many biologically important proteins, but have also been known to have powerful regulatory effects on cellular function [13,14], while amino acid transporters are primarily responsible for the translocation of amino acids by sensing the concentration of both extracellular and intracellular fluid. MeAIB has been used to elucidate the function of SNAT2 in human cells and significantly inhibits SNAT2 expression in IPEC-J2 cells that had 92% homology with human cells [4]. In the present study, we found that the addition of a 5 mM dose of MeAIB could better inhibit the SNAT2 that has more than 50% inhibition efficiency, while it has minimized the non-specificity that SNAT analogs and PAT1 show less than 20% inhibition efficiency compared with control group in this porcine cell line. The results suggest that suppression of SNAT2 in IPEC-J2 cells reduced cell proliferation, which was also supported by G1 arrest and the decrease in protein synthesis. Furthermore, we demonstrated that the cyclic AMP [15], an intracellular secondary messenger molecule involved in many signal transduction cascades, experienced a significant decrease in IPEC-J2 treated with MeAIB. In order to further explore the underlying reason, competitive inhibition of SNAT2 by MeAIB treatment led to a substantial reduction in the intracellular concentrations of neutral amino acids including key intermediary metabolites (glutamine, serine, and proline) as well as the indispensable amino acids methionine and leucine, which is consistent with previous reports that SNAT2—and not just SNAT2 substrates—also exerts an indirect effect in humans [2]. For l-alanine, the preferred naturally occurring substrate for SNAT2, there are no significant differences compared with MeAIB group. The reason is probably due to the lack of l-Ala in the culture medium. On the whole, the control group showing slightly higher levels might be due to the effect of 10% serum. We concluded that SNAT2 inhibition by the addition of MeAIB strongly impaired intracellular amino acid profiles and led to the inhibition of protein synthesis and cell growth.

The role of amino acid transporters in the regulation of the signaling pathway is now a well-established model in mammalian cells. The data in Figure 1 suggest that the level of SNAT2 inhibition had significant effects on mTOR phosphorylation. Such situations are likely attributable to the reduction of intracellular amino acids that could regulate the mTOR pathway by protein complexes composed of Rags, Ragulator, v-ATPase, GATOR, and folliculin [16,17,18]. Analysis of the genes related to the mTOR signaling pathway by an RT2 Profiler PCR Array system indicated that all 84 genes, including 21 differential genes, experienced just a slight change, 2.9-fold maximum, in the mRNA level. These results could suggest that total mRNA expression level may not play an important role in mTOR regulation in amino acid transporters because mTOR coordinated eukaryotic cell growth and metabolism with environmental inputs, including amino acid and growth factors, as primarily reflected by the state of phosphorylation, such as RAG GTPases for amino acids [19] and Tuberous Sclerosis Complex 1/2(TSC1/2) for growth factors [20]. When we further researched some of the differential genes with transcription factor function, we found that three mTOR-related genes with a negative effect, including *EIF4EBP1* [21], a translation repression protein, *IGFBP3* [22], which binds insulin-like growth factors with high affinity, and *DDIT4* [23], which is activated by certain stresses, have been upregulated, consistent with the inhibition of mTOR phosphorylation and cell growth. 

Although MeAIB was often used for a system A substrate to suppress the expression of SNAT2 in cells and study its function, there are some limitations on specificity, such as that MeAIB has been reported to inhibit other SNAT analogs and PAT1 [24,25]. The real-time PCR analysis of other transporters has provided a suitable concentration to research SNAT2, but it cannot totally clarify the SNAT2 function. Currently, we are trying to establish the SNAT2 knockout cell line and hope it will help us to further elucidate SNAT2 function. 

## 4. Materials and Methods

### 4.1. Antibodies and Reagents

Methylaminoisobutyrate (MeAIB) was purchased from Sigma. (^3^H)-Phenylalanine was provided by American Radiolabeled Chemical, Inc. (St. Louis, MO, USA); Rabbit anti-SNAT2, β-actin, p-mTOR, and mTOR were obtained from Cell Signaling Technology (Danvers, MA, USA).

### 4.2. Cell Culture and Treatment

IPEC-J2 were grown in a high-glucose Dulbecco’s modified Eagle medium (DMEM) (Hyclone, South Logan, UT, USA) containing 10% fetal bovine serum (Gibico, Gaithersburg, MD, USA) and 1% antibiotic solution (P/S) Sigma-Aldrich(St Louis, MO, USA) at 37 °C in a 5% CO_2_ incubator. After an overnight incubation, cells were incubated in a medium containing 0 or 5 mmoL/L System A amino acid analogue (MeAIB) for 48 h.

### 4.3. Cell Proliferation and Cycle Assays

Proliferation of IPEC-J2 was evaluated using a CCK-8 assay (Dojindo Laboratories, Kumamoto, Japan), based on the cleavage of the tetrazolium salt WST-8 by chondrial dehydrogenase in viable cells. After culture in 0 or 5 mmoL/L MeAIB medium for 2 h, 10 μL CCK-8 (5 mg/mL) was added to 96-well plates. After 2 h incubation at 37 °C, the absorbance at 450 nm of each well was measured using a Thermomax microplate reader.

For cycle assay, IPEC-J2 cells in a six-well plate were harvested after MeAIB treatment, washed with cold PBS, and fixed with ice-cold 70% ethanol for least 24 h. The cells were washed twice with PBS by centrifuging at 1500 rpm for 10 min, and then treated with 5 μg/mL RNase A (Sigma) for 1 h at 37 °C, stained with 25 µg/mL of propidium iodide (PI), and analyzed in a flow cytometer.

### 4.4. Determination of Protein Synthesis and Degradation

Protein synthesis was assayed by measuring the incorporation of (^3^H)-Phenylalanine into cell proteins as described previously [12]. IPEC-J2 cells in 10-cm dishes were incubated for 45 h in DMEM with MeAIB (0 or 5 mmoL/L), and then replaced with DMEM with (^3^H)-Phenylalanine (0.8 µCi per well, sp. act. 120–190 Ci/mmoL) for 3 h. Cells were washed with PBS three times and proteins were precipitated by the addition of ice-cold 2% (*v*/*v*) trichloroacetic acid for 10 min, followed by washing and incubation with methanol for 10 min. The cellular material was then solubilized in 1 M NaOH and incorporation of (^3^H)-Phenylalanine was quantified using liquid scintillation spectrometry.

For determining protein degradation, IPEC-J2 cells were cultured for 48 h in DMEM containing 0.1 mM l-phenylalanine plus (^3^H)-phenylalanine (0.8 µCi/well). After the 24-h culture to label cellular proteins, cells were washed three times with DMEM containing 1 mM l-phenylalanine and to deplete intracellular free (^3^H)-phenylalanine. The IPEC-J2 was then cultured for 3 h in 2 mL DMEM with 1 mM l-phenylalanine and MeAIB (0 or 5 mmoL/L). At the end of a 3-h culture period, both medium and cells were collected and determined as performed above. The percentage of protein-bound (^3^H)-phenylalanine released into culture medium ((^3^H)-phenylalanine in medium / (^3^H)-phenylalanine in cell proteins × 100) was calculated to indicate protein degradation in IPEC-1 cells.

### 4.5. Analysis of Intracellular Free Amino Acids, cAMP, and cGMP

IPEC-J2 cells were cultured in DMEM with MeAIB (0 or 5 mmoL/L) in 6-well platesfor 48 h. The cells were rapidly chilled on ice, rinsed three times with ice-cold 0.9% (*w*/*v*) NaCl, and collected by scraping. Amino acids were determined on an Agilent 1100 high-performance liquid chromatography system with Zorbax Eclipse AAA column (4.6 × 75 mm, 3.5 µm) at 40 °C with *o*-phthalaldehyde/3-mercaptopropionate/9-fluorenylmethyl chloroformate precolumn derivatization and ultraviolet and fluorimetric post column detection.

The Intracellular cAMP and cGMP were detected by immunoassay (Catalog #K371-100, Catalog #K372-100, BioVision, Milpitas, CA, USA) in accordance with the instructions.

### 4.6. Protein Extraction, SDS-PAGE, and Immunoblotting

IPEC-J2 cells were treated with MeAIB in six-well plates for 48 h. All samples were lysed for 10 min in ice-cold lysis buffer with a complete protease inhibitor cocktail. Immunoblotting assays were performed as described previously [26].

### 4.7. RT^2^ Profiler PCR Array Tests

A custom RT^2^ Profiler PCR Array (Qiagen, Hilden, Germany, Cat. no. 330231) was used to simultaneously detect numerous genes related to the mTOR signaling pathway. Total RNA was isolated from IPEC-J2 cells treated with MeAIB using TRIzol Reagent (Life Technologies, Carlsbad, CA, USA) and quantified with Nanodrop 2000 (Thermo Fisher Scientific, Waltham, MA USA). Total RNA (2 μg) was reverse-transcribed in a final volume of 20 µL with an RT^2^ First Strand Kit. The RT^2^ Profiler PCR Array tests were performed following the instructions of the manufacturer. The exported Ct values were input to a template Excel file provided by SABiosciences (Qiagen) and uploaded for online analysis. After data review, qualified data from 35 CMT and 5 NMGT samples were analyzed by applying the 2 ^−ΔΔ*C*t^ method.

### 4.8. Statistical Analysis

All data were expressed as mean ± standard deviation from at least three independent experiments. A one-way ANOVA with Tukey’s post hoc test was performed for analysis of the fold changes of genes in the RT^2^ Profiler PCR Array test results. A significant difference was expressed as * *p* < 0.05, while a highly significant difference was expressed as ** *p* < 0.01.

## 5. Conclusions

The major function of small-intestinal epithelium acts as digestion and absorption of nutrients, while amino acid transporters have a role in as sensors, as well as carriers, of tissue nutrient supplies. Understanding the relationship between intestinal nutrient absorption and intestinal amino acid transporters could help us to improve digestive systemfunction. Our work indicated that the role of SNAT2 in porcine intestinal epithelial cells was in translocating amino acids and supporting efficient proliferation and development in the mTOR pathway by regulating some transcription factors, which provide insight to nutritional regulation and therapy.

## Figures and Tables

**Table 1:** Intracellular free amino acid profile of IPEC-J2.

AA (μmol/mg Protein)	Control	MeAIB	*p*-Value
Alanine	0.956 ± 0.011	0.932 ± 0.011	0.169
Arginine	1.139 ± 0.035	1.049 ± 0.035	0.121
Asparagine	0.064 ± 0.004	0.034 ± 0.005	0.003
Glutamine	0.599 ± 0.033	0.105 ± 0.002	<0.001
Glutamate	1.704 ± 0.033	1.785 ± 0.076	0.365
Glycine	3.621 ± 0.098	1.571 ± 0.057	<0.001
Histidine	0.302 ± 0.007	0.123 ± 0.011	<0.001
Isoleucine	0.131 ± 0.015	0.343 ± 0.017	0.221
Leucine	0.946 ± 0.050	0.774 ± 0.036	0.031
Lysine	1.616 ± 0.023	1.686 ± 0.047	0.235
Methionine	0.061 ± 0.001	0.023 ± 0.003	<0.001
Phenylalanine	0.262 ± 0.018	0.260 ± 0.014	0.108
Proline	0.679 ± 0.040	0.254 ± 0.020	<0.001
Serine	2.693 ± 0.045	1.162 ± 0.048	<0.001
Threonine	0.859 ± 0.013	0.905 ± 0.033	0.244
Tryptophan	0.071 ± 0.003	0.078 ± 0.004	0.215
Tyrosine	0.025 ± 0.002	0.026 ± 0.003	0.719
Valine	0.135 ± 0.010	0.130 ± 0.011	0.74
Taurine	0.293 ± 0.023	0.301 ± 0.004	0.758

**Table 2:** Transcriptional expression profiles of mTOR signaling-related genes in IPEC-J2.

Genes	Fold Change	*p*-Value	The Role of Regulation	Description
*RPS6KA4*	1.651	0.042	mTOR Upstream Regulators for mTORC1 Positive Regulation	Ribosomal protein S6 kinase α-4-like
*DDIT4*	1.625	0.002	mTOR Upstream Regulators for mTORC1 Negative Regulation	DNA-damage-inducible transcript 4
*EIF4EBP1*	1.509	0.044	mTOR Downstream Effectors for mTORC1 Negative Regulation	Eukaryotic translation initiation factor 4E binding protein 1
*IGFBP3*	1.968	0.014	mTOR Upstream Regulators for mTORC1 Negative Regulation	Insulin-like growth factor binding protein 3
*INS*	1.875	0.034	mTOR Upstream Regulators for mTORC1 Positive Regulation	Insulin
*PIK3CG*	2.194	0.062	mTOR Upstream Regulators for mTORC1 and mTORC2 Positive Regulation	Phosphoinositide-3-kinase, catalytic, gamma polypeptide
*PRKCB*	2.179	0.01	mTOR Downstream Effectors for mTORC2 Positive Regulation	Protein kinase C, β
*RPS6KA1*	2.915	0.012	mTOR Upstream Regulators for mTORC1 and mTORC2 Positive Regulation	Ribosomal protein S6 kinase, 90 kDa, polypeptide 1
*RPTOR*	1.533	0.001		mTORC1 Complex
*ULK1*	1.58	0.002	mTOR Downstream Effectors for mTORC1 Negative Regulation	Unc-51-like kinase 1 (C. elegans)
*CAB39*	−1.95	0.001	mTOR Upstream Regulators for mTORC1 Negative Regulation	Calcium binding protein 39
*DDIT4L*	−1.905	0.02	mTOR Upstream	DNA damage-inducible transcript 4-like protein-like
*IRS1*	−2.561	<0.001	mTOR Upstream Regulators for mTORC1 Positive Regulation	Insulin receptor substrate 1
*KRAS*	−1.602	0.034	mTOR Upstream	GTPase KRas-like
*PIK3R2*	−1.598	0.011	mTOR Upstream	Phosphoinositide-3-kinase, regulatory subunit 2 (β)
*RPS6KA5*	−2.129	0.001	mTOR Upstream	Ribosomal protein S6 kinase, 90 kDa, polypeptide 5
*VEGFB*	−1.941	0.001	mTOR Downstream Effectors formTORC1 Positive Regulation	Vascular endothelial growth factor B-like
*RRAGA*	−1.790	0.002	mTOR Upstream Effectors for mTORC1 Positive Regulation	Ras-related GTP binding A
*VEGFC*	−1.628	0.012	mTOR Downstream Effectors for mTORC1 Positive Regulation	Vascular endothelial growth factor C-like
*YWHAQ*	−1.77	0.003	mTOR Upstream Regulators for mTORC1 Negative Regulation	Tyrosine 3-monooxygenase/tryptophan 5-monooxygenase activation protein, theta polypeptide

## Abbreviations

SNAT2	Sodium-coupled neutral amino acid transporter 2
mTOR	Mammalian target of rapamycin
IPEC	Intestinal porcine epithelial cells
MeAIB	System A amino acid analogue
DMEM-H	High-glucose Dulbecco’s modified Eagle’s
cAMP	Cyclic adenosine monophosphate
cGMP	Cyclic guanosine monophosphate
